# Erythroblastic Islands in the Bone Marrow of Patients with Immune-Related Pancytopenia

**DOI:** 10.1371/journal.pone.0095143

**Published:** 2014-04-16

**Authors:** Yi-Hao Wang, Rong Fu, Shu-Wen Dong, Hui Liu, Zong-Hong Shao

**Affiliations:** Department of Hematology, Tianjin Medical University General Hospital, Tianjin, China,; National Institutes of Health, United States of America

## Abstract

**Background:**

Immune-related pancytopenia (IRP) is characterized by pancytopenia caused by autoantibody-mediated bone marrow destruction or suppression. The bone marrows of IRP patients have remarkably increased erythroblastic islands (EIs).

**Methodology and Principal Findings:**

We determined the immunoglobulin G (IgG) autoantibodies in some parts of EIs of IRP patients using immunofluorescence to investigate the biological function of EIs with IgG in the pathophysiology of IRP. The dominant class of autoantibodies detected in mononuclear cells was IgG (CD34 IgG, CD15 IgG, and GlycoA IgG), specifically IgG on GlycoA-positive cells (GlycoA IgG). Results show that extravascular hemolysis occurred in IRP through IgG autoantibodies in the EIs. These data included a high percentage of reticulocytes in the peripheral blood, hypererythrocytosis in the bone marrow, and high serum bilirubin. Furthermore, we examined the macrophages in the bone marrow of IRP patients. The results show that the number of activated macrophages relatively increased, and the phagocytic activity of macrophages significantly increased.

**Conclusions and Significance:**

Increased EIs with IgG were the sites of erythroblast phagocytosis by the activated macrophages, rather than erythropoietic niches. The IgG autoantibodies in the EIs possibly functioned as adhesion molecules for a ring of erythroblasts around the macrophages, thereby forming morphologic EIs.

## Introduction

Immune-related pancytopenia (IRP) is a form of bone marrow failure caused by hematopoiesis-suppressing autoantibodies. The clinical characteristics of patients with IRP include cytopenia or pancytopenia, normal or high reticulocyte (Ret) and neutrophil counts, hypoplastic or hyperplastic bone marrow, increased erythroblastic islands (EIs), and erythroid hyperplasia [1]. Redundant autoantibodies on bone marrow mononuclear cells (BMMNCs) can be detected in the bone marrow by BMMNC–Coombs test or flow cytometry (FCM).

EIs are distinct anatomical units that consist of a central macrophage surrounded by a ring of developing erythroblasts, and are considered functional units of definitive erythropoiesis [2]–[8]. Macrophages and erythroblasts maintain the integrity of EIs through several adhesion molecules. Macrophages participate in the terminal maturation of erythroblasts by functioning as adhesion molecules, providing nutrients, transmitting proliferative and survival signals, and inducing the enucleation of erythroblasts [9]–[18]. However, these macrophage functions have not been directly confirmed.

IRP patients have more EIs in their bone marrow, which suggests that erythropoietic cells undergo hyperplasia in the bone marrow [2]–[8]. These patients also experience varying degrees of anemia, which are worse in severe cases. In addition, more erythroblasts are phagocytosed by macrophages in the bone marrow of IRP patients with increased EIs. Immunosuppression therapy with prednisolone and human immunoglobulin decreases the autoantibodies in BMMNCs to undetectable levels. The number of EIs return to normal, and the phagocytosis of erythroblasts by macrophages ceases. Moreover, the degree of anemia is ameliorated. Information on the ineffective hematopoiesis of increased EIs in IRP patients is limited.

This study aimed to elucidate the significance of increased EIs in the bone marrow of IRP patients. Thus, we investigated the erythroblast autoantibodies, biological characteristics of macrophages, and clinical characteristics of IRP patients.

## Materials and Methods

### Ethics

This study complied with international regulations concerning the ethical participation of volunteers. The protocol used in this study and signed informed consent forms of the participants were approved by the Ethics Committee of the General Hospital of TianJin University, China.

### Patients

The participants were diagnosed with IRP according to the published criteria [1]. Patients who suffered severe systemic infections that could not be controlled by antibiotics or those with viral infections, such as hepatitis viruses, cytomegalovirus, human parvovirus B, or Epstein–Barr virus, were excluded from the study.

The following diseases were also ruled out. Myelodysplastic syndrome was excluded by a morphological assay, BMMNC culture, karyotyping, conventional cytogenesis and fluorescence in situ hybridization, as well as genetic and immunophenotyping tests. CD55 and CD59 were detected in the granulocytes and bone marrow cells of patients using FCM. A flare assay was performed to exclude patients with paroxysmal nocturnal hemoglobinuria. Patients with hemophagocytic syndrome (HPS) were excluded based on the following clinical characteristics: persistent high fever; enlarged lymph nodes, spleen, and liver; high serum lactic dehydrogenase (>1000 U L^−1^) and ferritin; high levels of liver transaminase; and hyperlipidemia. Chromosomal breakage was analyzed to rule out Fanconi anemia among patients manifesting clinical characteristics compatible with this diagnosis.

All patient samples were obtained before therapy or after recovery and discontinuation of therapy for more than six months.

### Evaluation of Responses to Treatment

Patients who participated in the treatment demonstrated increased blood count within three months of treatment. The quality of their responses was analyzed at three and six months of treatment according to the following criteria. (1) Essential cure was observed when: the hemoglobulin levels of male patients reached 120 g L^−1^ and those of female patients reached 110 g L^−1^, their granulocyte counts reached ≥1.5×10^9^ L^−1^, their platelet counts reached ≥100×10^9^ L^−1^ without suffering anemia and hemorrhage; and no relapse was observed during the one-year follow-up period. (2) Remission, defined as the absence of anemia and hemorrhage, was characterized by blood cell levels reaching the baseline (hemoglobulin count of 120 g L^−1^ for males and 110 g L^−1^ for females), an increase in granulocyte count of at least 3.0×10^9^ L^−1^, and an increase in platelet count of at least 30×10^9^ L^−1^ within one year. (3) Remarkable improvement, defined as the alleviation of anemia and hemorrhage, was characterized by an increase in hemoglobulin count of at least 30 g L^−1^ for at least three months, and the patient no longer requiring blood transfusion. (4) No response was defined as the patient not showing any improvement after completing the treatment.

### Treatment Protocol

All IRP patients were treated with immunosuppressive agents, namely, glucocorticoid alone or high-dose immunoglobulin (HDIVIG). Prednisolone was administered to all patients at a single oral dose of 0.5 to 1.0 mg kg^−1^ body weight d^−1^. Human immunoglobulin was infused at 0.4 g kg^−1^ body weight d^−1^ for 1 d to 5 d, followed by 10.0 g once a week for one to three months.

The patients were prescribed supportive care, including component transfusion, liver protection, jaundice therapy, calcium supplementation, and suppression of gastric acid secretion.

Smears of the initial bone marrow aspirates were examined under Wright's stain and cytochemical stains. The peripheral blood cell counts of patients were measured using an automatic hemocyte analyzer. The severity of anemia was stratified based on the hemoglobin concentrations: mild anemia, ≥90 g L^−1^; moderate anemia, 60 g L^−1^ to 90 g L^−1^; severe anemia, 30 g L^−1^ to 60 g L^−1^; and very severe anemia, ≤30 g L^−1^. Counts of serum bilirubin, triglycerides, ferritin, fibrinogen, and albumin were determined using routine laboratory methods.

### Immunofluorescence (IF) Assay

Cells were fixed in ice-cold ethanol and washed three times with PBS for 3 min.

The cells on the cover slips were blocked with 0.1% Triton X-100 and H_2_O_2_ for 6 min, rinsed with distilled water, and washed three times with PBS for 3 min. The cells were then blocked with rabbit serum for 10 min. The residual serum was removed, and the remaining content was incubated overnight with FITC-conjugated anti-hIgG at 4°C. The cells were rinsed three times with PBS for 3 min each, and stained with hematoxylin for 1 min. The stained cells were washed with diluted ammonia water and rinsed two times for 3 min. The prepared specimen was sealed with glycerol and examined under a fluorescence microscope.

### Macrophage Phagocytosis of Chicken Red Blood Cells (CRBCs)

#### 1. Preparation of macrophages

Bone marrow aspirates (5 mL) were collected in the evacuated tubes containing heparin as an anticoagulant. The BMMNCs were separated from the bone marrow aspirates using 75% Ficoll–Hypaque suspended in RPMI-1640 (Gibco-BRL, Grand Island, NY, USA) without sterile fetal bovine serum (FBS) in a 75 mL culture bottle. The BMMNCs were then incubated for 4 h at 37°C under a saturated 5% CO_2_ atmosphere. The macrophages that adhered onto the bottle wall and the suspended non-adherent cells were discarded. The adherent cells were digested with 0.25% pancreatin containing 0.01% Ethylene Diamine Tetraacetie Acid (EDTA). The cells were cultured in LG-DMEM culture solution containing 10% FBS. The supernatant was removed and the cells were harvested using 0.2% trypsase solution. The BMMNCs were resuspended in RPMI-1640 and then stored for future use.

#### 2. Preparation of CRBCs

Venous blood (1 mL) was drawn into a sterile syringe containing Alsever's solution. The CRBCs were washed with sterile saline three times to remove the white blood cells and platelets.

#### 3. Macrophage phagocytosis of CRBCs

The BMMNCs were infused with 1 mL of CRBCs and incubated for 1 h (shaken every 15 min). After the cell suspension was centrifuged at 10,000 rpm for 10 min and 1000 rpm for 5 min, the supernatant was removed. Cell smears were prepared by fixing the cells with methanol/formaldehyde solution and staining them with Giemsa. A total of 100 macrophages were examined under a microscope.

The phagocytic ratio and index were calculated as follows: phagocytic ratio  =  number of macrophages that phagocytosed CRBCs/total number of macrophages; phagocytic index  =  number of CRBCs phagocytosed by macrophages/total number of macrophages.

### Preparation of Bone Marrow Samples and FACS Analysis

Bone marrow aspirate (2 mL) was collected in a heparinized tube and passed through a 0.2 µm membrane filter (Nuclepore) to remove cellular debris. Approximately 50 µL of bone marrow aspirate was transferred to a 5 mL tube, and incubated with monoclonal antibodies. FITC/PE/APC–conjugated GlycoA/CD15/CD34 (20 µL; Becton Dickinson) was added into the aspirate, and the mixture was incubated for 30 min at 2°C to 8°C. RBC lysis solution was used to eliminate the RBCs from the whole bone marrow aspirate. The cell suspension was centrifuged at 1500 rpm for 5 min. The resulting supernatant was discarded. PBS buffer (5 mL) was added, and the resulting mixture was centrifuged at 1500 rpm for 5 min. The supernatant was discarded, and the cells were washed two times. The cells were resuspended in 200 µL of PBS buffer and fixed with formaldehyde for FACS analysis. The cells were placed in a separate tube as the control sample, and treated with FITC/PE/APC–labeled mouse IgG1 antibodies. Approximately 50,000 events were selected in the Acquisition tab of CellQuest software to record the field of specimen tubes in FACSArial (Becton Dickinson).

### Statistical Analysis

Measurement data, including the counts of Ret, EI, hemoglobulin, TBIL, DBIL, erythroid in the bone marrow, and macrophages, were presented as means ± standard deviations and analyzed using ANOVA. The differences in IgG/IgM-positive rates and treatment efficiency between groups were analyzed using chi-square test. Differences with p<0.05 were considered significant. Statistical analysis was performed using SAS PLINK v.1.07.

## Results

### Patient Characteristics

A total of 81 patients diagnosed with IRP who were admitted into the Department of Hematology of Tianjin General Hospital from August 2008 to December 2011 were enrolled in this study. The ages of patients ranged from 4 years to 74 years, and the median age was 29 years. Among the 81 patients, 39 were male and 42 were female. Twenty patients (13 males and seven females) had severe aplastic anemia (SAA) but were left untreated (aged 14 years to 62 years with a median age of 33 years). Fifteen healthy volunteers (aged 11 years to 76 years) were included as controls (nine males and six females). The groups did not significantly differ in age and sex distribution (Table 1).

**Table 1:** Characteristics of the study groups.

CHARACTERISTICS	IRP GROUP	SAA GROUP	HEALTHY GROUP	p value
No. of patients	81	20	15	
Sex (M/F)	39/42	13/7	9/6	
Age (years)	29 (4 to 74)	33 (14 to 62)	36 (11 to 76)	
Ret (%)	1.46±0.60	1.23±0.45	0.15±0.13	<0.0001^‡¥^
EI	7.81±3.78	0.10±0.31	4.00±1.46	<0.0001^‡¥^
EI with positive IF (no. of cases)	30	0	0	
EI with negative IF (no. of cases)	51	20	15	

Values are presented as median; ranges are shown in parentheses.

Values are representative pre-transfusion counts obtained at the time of treatment.

¥ IRP group compared with SAA group; ^‡^ IRP group compared with healthy group.

### EIs in the Bone Marrow Aspirates of IRP Patients

**Figure 1:**
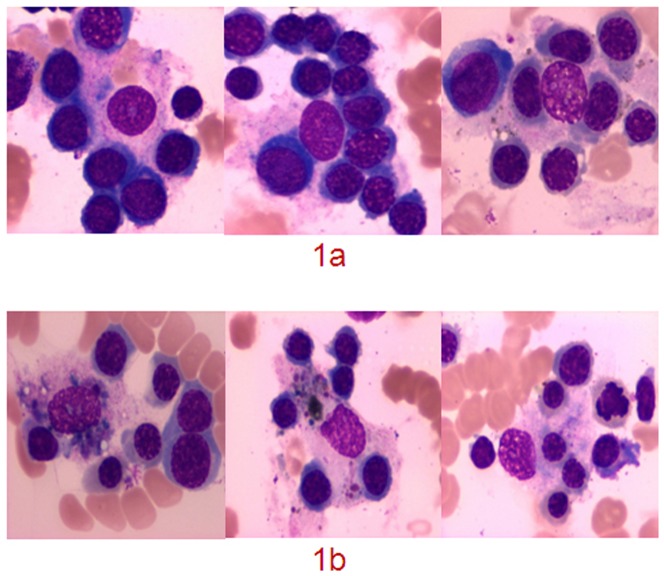
EIs and erythroblasts phagocytosed by macrophages. (1a) EIs in bone marrow aspirates of patients with IRP (Wright–Giemsa, ×100); (1b) Erythroblasts phagocytosed by macrophages in the bone marrow aspirates of patients with IRP (Wright–Giemsa, ×100).

EIs accompanied by macrophage-phagocytosed erythroblasts were easily observed in the bone marrow aspirates of IRP patients (Figure 1). The IRP patients, SAA patients, and healthy controls had 7.81±3.78, 0.10±0.31, and 4.00±1.46 EIs, respectively, in every bone marrow aspirate smear (2.0 cm×1.5 cm). The IRP patients, SAA patients, and healthy volunteers significantly differed in terms of EI count (p<0.0001; Table 1).

### Ret in the Peripheral Blood of IRP Patients

The IRP patients did not significantly differ from the healthy controls in terms of the percentage of Ret in the peripheral blood (1.46±0.60 vs. 1.23±0.45) (p = 0.113; Table 1). The percentage of Ret in the SAA patients was 0.15±0.13, which was lower than those of the IRP patients and healthy controls (p<0.0001).

### IF Detection of IgG Autoantibodies in the Bone Marrow Aspirate Smears in IRP Patients

**Figure 2:**
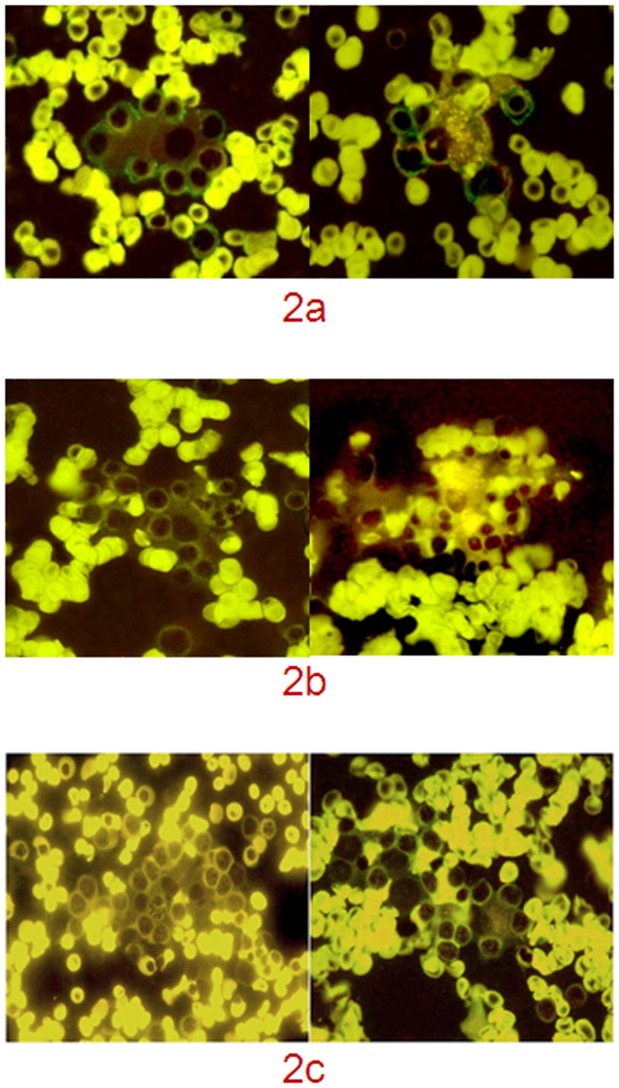
IgG autoantibodies detected by IF in EIs. (2a) IgG autoantibodies detected by IF in EIs of patients with IRP (IF and hematoxylin, ×100); (2b) No IgG autoantibodies detected by IF in EIs of patients with IRP (IF and hematoxylin, ×100); (2c) EIs of healthy groups (IF and hematoxylin, ×100).

Intense green fluorescence was observed in the EIs of IRP patients, particularly between the macrophages and erythroblasts. This result indicates that the IgG autoantibodies aggregated in the EIs, specifically at the junction between macrophages and erythroblasts. The results show that 30 of the 81 IRP patients were positive for IgG autoantibodies in their EIs (positive IF of EIs). By contrast, no IgG autoantibodies (negative IF of EIs) were observed in the EIs of the SAA patients and healthy controls (Figure 2).

### Clinical Characteristics of IF-Positive EIs in IRP Patients

The IF-positive IRP patients had significantly more EIs than the IF-negative IRP patients and healthy controls (11.13±3.20 vs. 5.86±2.53, p<0.0001; and 11.13±3.20 vs. 4.00±1.46, p<0.0001, respectively). The EIs of the IRP patients decreased after treatment. The number of EIs in the IRP patients with IF-positive EIs before treatment was significantly higher than that after treatment (11.13±3.20 vs. 4.33±1.86, p<0.0001), but did not significantly differ between the treated IRP patients and healthy controls (4.33±1.86 vs. 4.00±1.46, p = 0.516).

The 30 IRP patients with IF-positive EIs (22 with severe anemia and eight with moderate anemia) had a median hemoglobulin concentration of 54.87±8.0 g L^−1^. The 51 IRP patients with IF-negative EIs (16 with severe anemia and 26 with moderate anemia) had a median hemoglobulin concentration of 63.63±8.31 g L^−1^ (Table 2).

**Table 2:** Characteristics of IRP patients with positive IF in EI groups.

CHARACTERISTIC	Patients with IRP exhibiting positive IF of EI	Patients with IRP exhibiting negative IF of EI	p value
No. of patients	30	51	
Sex (M/F)	14/16	25/26	
Age (year)	32 (12 to 63)	28 (4 to 74)	
EI	11.13±3.20	5.86±2.53	<0.0001
Ret (%)	1.91±0.64	1.19±0.37	<0.0001
IgG autoantibodies	30	38	
GlycoA IgG autoantibodies	29	2	
Hemoglobulin (g L^−1^)	54.87±8.06	63.63±8.31	<0.0001
Severity of anemia			
mild	0	0	
moderate	8	26	
sever	22	16	
Very sever	0	0	
TBIL (mmol L^−1^)	16.75±3.99	22.34±5.92	<0.0001
DBIL (mmol L^−1^)	10.11±1.62	16.03±4.40	<0.0001
Percentage of erythroid in the bone marrow	34.11±5.22	24.64±4.95	<0.0001
Percentage of macrophages	0.62±0.02	0.52±0.07	<0.0001
Percentage of activated macrophages	44.36±5.09	34.85±7.47	<0.0001
Phagocytic rate of macrophages	40.77±8.43	35.25±7.34	0.004
Phagocytic index of macrophages	0.73±0.09	0.58±0.12	<0.0001

TBIL, total serum bilirubin.

IBIL, serum indirect bilirubin.

Values are presented as median; ranges are shown in parentheses.

Values are representative pre-transfusion counts obtained at the time of treatment.

The percentage of Ret in the IRP patients with IF-positive EIs was significantly higher than that in the IRP patients with IF-negative EIs (1.91±0.64 vs. 1.19±0.37, p<0.0001).

**Figure 3:**
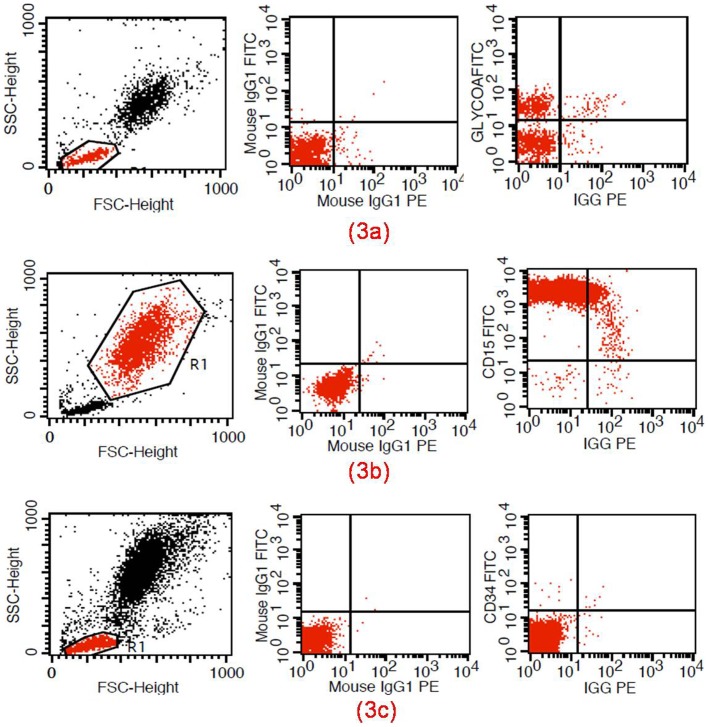
Detection of autoantibodies to BM hemopoietic cells. (3a) Autoantibodies detected on nucleated erythrocytes(GlycoA^+^); (3b) Autoantibodies detected on granulocytes(CD15^+^); (3c) Autoantibodies detected on stem cells(CD34^+^).

During the detection of autoantibodies on BMMNCs by FACS, the FSC/SSC gate attracted the populations of nucleated erythrocytes, stem cells, and granulocytes. The fluorescence data from events in the gate were analyzed to determine the percentages of various subpopulations (CD34^+^, CD15^+^, and GlycoA^+^) (Figure 3). The IgG autoantibodies of the 30 IRP patients with IF-positive EIs tested positive for IgG autoantibodies (CD34 IgG, CD15 IgG, or GlycoA IgG). GlycoA IgG was detected in 29 of the 30 patients. Although 38 of the 51 IRP patients with IF-negative EIs also showed IgG autoantibodies, only two were positive for GlycoA IgG (Table 2). No autoantibodies (IgG/IgM) were detected in all the control groups.

The IRP patients with IF-positive EIs showed high levels of total serum bilirubin (22.34±5.92 µmol L^−1^) and indirect bilirubin (16.03±4.40 mmol L^−1^), which were higher than those of the IRP patients with IF-negative EIs (16.75±3.99 µmol L^−1^ and 10.11±1.62 µmol L^−1^, respectively).

The percentage of erythroid lineage cells in the bone marrow aspirates of IRP patients with IF-positive EIs were significantly higher than that of IRP patients with IF-negative EIs (34.11±5.22 vs. 24.64±4.95, p<0.001). This result suggests that compensatory erythroid hyperplasia occurred in IRP patients.

The IRP patients with IF-positive EIs had more macrophages in their bone marrow aspirates (0.62±0.02 of IF was activated) than the IRP patients with IF-negative EIs. The IRP patients with IF-positive EIs had more activated macrophages than the IRP patients with IF-negative EIs (0.62±0.02 vs. 0.52±0.07, p = 0.004) and healthy controls (44.36±5.09 vs. 34.85±7.47, p<0.0001). Phagocytosis of CRBCs improved the biological function of macrophages. Moreover, the phagocytic ratio and index were significantly higher among the IRP patients with IF-positive EIs than those among the IRP patients with IF-negative EIs (40.77±8.43 vs. 35.25±7.34, p = 0.004; 0.73±0.09 vs. 0.58±0.12, p<0.0001, respectively).

### IRP Patient Responses to Treatment

All patients received the glucocorticoid. Moreover, 22 cases of IRP patients with IF-positive EIs and 34 cases of IRP patients with IF-negative EIs simultaneously received HDIVIG. Three months after the initial treatment, the IRP patients with IF-positive EIs exhibited a total treatment efficiency of 56.7% positive IF of EIs (6.7% experienced essential treatment, 13.3% experienced remission, and 36.7% experienced remarkable improvement). By contrast, the IRP patients with IF-negative EIs exhibited lower treatment efficiency (p<0.05). The IRP patients with IF-positive EIs exhibited significantly improved total efficiency (86.7% vs. 62.75%) compared with the IRP patients with IF-negative EIs (Table 3).

**Table 3:** Response of IRP patients to treatment.

Group	Cases	Three months					Six months				
		Essential treatment	Remission	Remarkable improvement	No response	Total efficiency	Essential treatment	Remission	Remarkable Improvement	No response	Total efficiency
A	30	2 (6.7%)	4 (13.3%)	11 (36.7%)	13 (43.3%)	17 (56.7%)	5 (16.7%)	9 (30.0%)	12 (40.0%)	4 (13.3%)	26 (86.7%)
B	51	1 (1.9%)	5 (9.8%)	13 (25.4%)	32 (62.7%)	19 (37.3%)	6 (11.7%)	7 (13.7%)	21 (41.1%)	17 (33.3%)	32 (62.7%)

A, IRP with positive IF of EI; B, IRP with negative IF of EI.

## Discussion

In 2000, Shao first detected autoantibodies in the BMMNCs (bone marrow hematopoietic stem cells, nucleated erythrocytes, and granulocytes) in IRP patients using a BMMNC–Coombs test [19]. These autoantibodies are produced by hyperfunctional B lymphocytes [20]–[22]. IgG autoantibodies activate macrophages, thereby causing the phagocytosis of hematopoietic cells [23]. IgM autoantibodies activate the complement system [24], which causes hematopoietic cell lysis. These autoantibodies bind to membrane functional antigens, such as erythropoietin receptors [25]. These patients are diagnosed with IRP, which is also known as “positive BMMNC–Coombs test pancytopenia” [26].

The IRP patients had more EIs in their bone marrow than the healthy controls. Whether the increase in EIs in these IRP patients are relevant to autoantibodies should be confirmed. Thus, we detected autoantibodies in EI. In the beginning of this study, laser confocal microscopy was used to observe the result of autoantibodies. The preliminary experimental results were not ideal even though FITC-conjugated goat anti-human IgG and DAPI-stained cell nuclei were observed. However, the nucleic area of each nucleated cell exhibited bright blue fluorescence, and the cell contour, nucleus structure and contour, and nucleus/plasma ratio were not evident. Thus, single nucleated cells (immature granulocytes, nucleated erythrocytes and lymphocytes, macrophages, or monocytes) were harder to identify. Furthermore, the EIs (nucleated erythrocytes at various developmental stages surrounding macrophages) and aggregated multiple nucleated cells (aggregated structure of nucleated erythrocytes, immature granulocytes, or mixed immature nucleated cells) were difficult to distinguish. Other novel techniques were used to stain the nuclei to distinguish the single nucleated cells, and verify that this technique did not affect the results of the IF assay. The cell nuclei were stained using Wright–Giemsa. However, the autofluorescence of Wright–Giemsa was so intense that it interfered with the results of the IF assay. We then stained the nuclei with hematoxylin, which obtained surprisingly good results. The green fluorescence of the FITC-labeled autoantibodies was visible, allowing us to distinguish the nucleated cells and identify the EIs, which consisted of nucleated erythrocytes at various stages that surrounded macrophages. Hematoxylin exhibited spontaneous fluorescence that did not interfere with the results of the study. Hematoxylin stained the nucleus but not the cell membrane. However, the FITC-labeled IgG autoantibodies were localized on the cell membrane of nucleated erythrocytes and/or membrane junction between the macrophage and nucleated erythrocytes. The cells were clearly distinguishable because of the significant difference in position and cell morphology. Thus, hematoxylin autofluorescence did not interfere with the results of our study. The IF micrographs showed the phagocytosis of erythroblasts by macrophages, not the overlapping structures. If overlapping structures were present, green fluorescence was observed in the cytoplasm or nucleus or exhibited a disorderly distribution.

IgG autoantibodies were detected in the EIs of IRP patients, and these IgG autoantibodies primarily aggregated at the junction between macrophages and erythroblasts in the EIs. This result suggests that IgG autoantibodies functioned as adhesion factors between erythroblasts and macrophages in the EIs. We analyzed the clinical characteristics of the IRP patients with IF-positive EIs to understand the effects of EIs with IgG autoantibodies on the pathogenesis of IRP. The total number of EIs in bone marrow aspirate smears of the IRP patients with IF-positive EIs was significantly higher than those of the IRP patients with IF-negative EIs (11.13±3.20 vs. 5.86±2.53, p<0.0001) and healthy controls (11.13±3.20 vs. 4.00±1.46, p<0.0001). These EIs were also accompanied by macrophage-phagocytosed erythroblasts (Figure 1). HPS was not observed if the lymph nodes, spleen, and liver were not enlarged. Hyperlipidemia, high ferritin level, and persistently high fever were also not observed. IF detected IgG autoantibodies (CD34/CD15/GlycoA in all 30 patients and GlycoA IgG in 29/30 patients) in the erythroblasts of BMMNC-Ab of the IRP patients. Although IgG autoantibodies were also detected in the IRP patients with IF-negative EIs, these patients had less GlycoA IgG in their erythroblasts. Extravascular hemolysis was also observed in the IRP patients with IF-positive EIs, which resulted in severe anemia, increased percentage of Ret in the peripheral blood, increased number of erythroid lineage cells in the bone marrow, and increased TIBIL and IBIL levels. However, the Coombs test of the peripheral blood was negative, which excluded the possibility of autoimmune hemolytic anemia. This result indicates that erythroid hemolysis occurred in the bone marrow. We also determined the biological characteristics of macrophages in the bone marrow of IRP patients to understand the molecular interactions between erythroblasts and macrophages in the EIs. The IRP patients with IF-positive EIs had more macrophages in their bone marrow than the IRP patients with IF-negative EIs (34.11±5.22 vs. 24.64±4.95, p<0.0001). Most of these macrophages were activated (0.62±0.02). We examined the biological function of macrophages using an in vitro CRBC co-culture. The IRP patients with IF-positive EIs exhibited a higher ratio and index of macrophages than the IRP patients with IF-negative EIs. Thus, macrophages were activated by several factors that enhanced phagocytosis. The macrophages in the bone marrow were possibly activated by IgG autoantibodies, particularly GlycoA IgG on the erythroblasts. The Fc receptors on the macrophages could bind to the Fc regions of the erythroblast IgGs. Thus, the erythroblasts with IgG autoantibodies were recruited and surrounded by activated macrophages to form a morphologic EI structure. The IgG autoantibody-mediated activation of the macrophage-phagocytosed adjacent erythroblasts caused extravascular hemolysis. These EIs with the IgG autoantibody were possibly caused by early biological processes, in which erythroblasts with the IgG autoantibody were phagocytosed by activated macrophages, not the erythropoietic niches.

We also investigated the responses of the IRP patients with IF-positive EIs to HDIVIG or glucocorticoid treatments. The IRP patients with IF-positive EIs exhibited higher total treatment efficacies after three and six months of treatment than the IRP patients with IF-negative EIs. BMMNC-Ab was inhibited and removed after glucocorticoid treatment. The treatment also removed the autoantibodies in the EIs of IRP patients and decreased the number of morphologic EIs in the bone marrow smears, and the treated IRP patients did not significantly differ from the healthy controls (4.33±1.86 vs. 4.00±1.46, p<0.0001). The treatment also removed the macrophage-phagocytosed erythroblasts, which implies that EIs with IgG autoantibodies consisted of a central activated macrophage and a ring of erythroblasts with IgG autoantibodies. The formation and integrity of the EI structure was mediated by the IgG autoantibodies. Blocking the FcR receptor on macrophages with HDIVIG or inhibiting autoantibody production through glucocorticoid treatment resulted in the following: (1) formation and maintenance of EIs that lack the essential adhesion factors (IgG and/or FcR); (2) non-activation of macrophages, which did not recruit erythroblasts; and (3) disappearance of EIs with IgG autoantibodies.

These results show that the morphologic EIs in the bone marrow of IRP patients were not all erythropoietic sites. EIs with IgG autoantibodies in IRP patients were sites wherein erythroblasts were phagocytosed by macrophages. These autoantibodies could function as adhesion factors that attach erythroblasts to macrophages to form EIs. Thus, these EIs were the early biological markers of bone marrow failure in the pathogenesis of IRP.

## References

[1] Yue LZ, Shao ZH (2012) Research progress on the red cell diseases in China. Chin Med J(Engl) 125: 2746–2751. 22931985

[2] Yokoyama T, Etoh T, Kitagawa H, Tsukahara S, Kannan Y (2003) Migration of erythroblastic islands toward the sinusoid as erythroid maturation proceeds in rat bone marrow. J Vet Med Sci 65: 449–452. 12736425 10.1292/jvms.65.449

[3] Chasis JA (2006) Erythroblastic islands: specialized microenvironmental niches for erythropoiesis. Curr Opin Hematol 13: 137–141. 16567955 10.1097/01.moh.0000219657.57915.30

[4] Manwani D, Bieker JJ (2008) The erythroblastic island. Curr Top Dev Biol 82: 23–53. 18282516 10.1016/S0070-2153(07)00002-6 PMC3234703

[5] Chasis JA, Mohandas N (2008) Erythroblastic islands:niches for erythropoiesis. Blood 112: 470–478. 18650462 10.1182/blood-2008-03-077883 PMC2481536

[6] Gerber B, Nair G, Stussi G (2010) Erythroblastic islands. Br J Haematol 150: 499. 20560969 10.1111/j.1365-2141.2010.08260.x

[7] Astwood E, Vora A (2011) Erythroblastic islands. Blood 117: 10. 21265064 10.1182/blood-2009-05-207175

[8] Socolovsky M (2013) Exploring the erythroblastic island. Nat Med 19: 399–401. 23558622 10.1038/nm.3156

[9] Lee G, Lo A, Short SA, Mankelow TJ, Spring F, et al (2006) Targeted gene deletion demonstrates that cell adhesion molecule ICAM-4 is critical for erythroblastic island formation. Blood 108: 2064–2071. 16690966 10.1182/blood-2006-03-006759 PMC1895542

[10] Soni S, Bala S, Gwynn B, Sahr KE, Peters LL, et al (2006) Absence of eryhroblast macrophage protein (Emp) leads to failure of erythroblast nuclear extrusion. J Biol Chem 281: 20181–20189. 16707498 10.1074/jbc.M603226200

[11] Soni S, Hanspal M (2006) Cellular (2006) Localization and Developmental Expression of Emp Correlates with its Role in Erythroblastic Island Formation. Blood (ASH Annual Meeting Abstracts) 108: 537.

[12] Fabriek BO, Polfliet MM, Vloet RP, van der Schors RC, Ligtenberg AJ, et al (2007) The macrophage CD163 surface glycoprotein is an erythroblast adhesion receptor. Blood 109: 5223–5229. 17353345 10.1182/blood-2006-08-036467

[13] Fischer S, Kurbatova P, Bessonov N, Gandrillon O, Volpert V, et al (2012) Modeling erythroblastic islands: using a hybrid model to assess the function of central macrophage. J Theor Biol 298: 92–106. 22245622 10.1016/j.jtbi.2012.01.002

[14] Chow A, Huggins M, Ahmed J, Hashimoto D, Lucas D, et al (2013) CD169(+) macrophages provide a niche promoting erythropoiesis under homeostasis and stress. Nat Med 19: 429–436. 23502962 10.1038/nm.3057 PMC3983996

[15] Mao X, Shi X, Liu F, Li G, Hu L (2013) Evaluation of erythroblast macrophage protein related to erythroblastic islands in patients with hematopoietic stem cell transplantation. Eur J Med Res 118: 9. 10.1186/2047-783X-18-9 PMC3637484 23566571

[16] Soni S, Bala S, Kumar A, Hanspal M (2007) Changing pattern of the subcellular distribution of erythroblast macrophage protein (Emp) during macrophage differ entiation. Blood Cells Mol Dis 38: 25–31. 17071116 10.1016/j.bcmd.2006.09.005 PMC1857287

[17] Kurenkov EL, Kuznetsov ME, Rassokhin AG, Zakharov IuM (2006) The content of Ribonucleopro-teids in the bone marrow erythroblastic islands in various functional states of erythropoiesis. Ross Fiziol Zh Im I M Sechenova 92: 1339–1344. 17385426

[18] Mohandas N, Chasis JA (2010) The erythroid niche: molecular processes occurring with in erythroblastic islands. Transfus Clin Biol 17: 110–111. 20655267 10.1016/j.tracli.2010.05.009 PMC3684560

[19] He H, Shao ZH, Cao Z (2000) Category of bone marrow mononuclear cells-Coombs test. Zhonghua Xue Ye Xue Za Zhi 21: 550.

[20] Fu R, Shao ZH, Liu H, Wu YH, Wang HQ, et al (2007) Role of B lymphocyte and its subpopulations in pathogenesis of immunorelated pancytopenia. Chin Med Sci J 22: 199–202. 17966171

[21] Fu R, Shao ZH, Liu H, He H, Jia HR, et al (2003) Category, quantity and clinical significance of autoantibodies on bone marrow hematopoietic cells in patients with immunorelated cytopenia. Zhonghua Xue Ye Xue Za Zhi 24: 177–180. 12864946

[22] Fu R, Shao ZH, He H, Liu H, Jia HR, et al (2002) Quantity and apoptosis-related protein level of B lymphocyte in patients with immunorelated pancytopenia. Zhonghua Xue Ye Xue Za Zhi 23: 236–238. 12133443

[23] Wang YH, Fu R, Shao ZH, Wang HQ, Xing LM, et al (2009) Study on quantity and function of bone marrow macrophages in patients with BMMNC-Coombs Test(+) pancytopenia. Zhonghua Xue Ye Xue Za Zhi 30: 538–542. 19954642

[24] Chen J, Fu R, Li LJ, Liu H, Wang YH, et al (2009) Variation in complement level and its significance in cytopenia patients with positive BMMNC-Coombs. Zhonghua Xue Ye Xue Za Zhi 30: 454–457. 19954597

[25] Liu H, Fu R, Wang YH, Liu H, Li LJ, et al.. (2013) Detection and analysis of autoantigen targeted by autoantibodies in Immuno-related Pancytopenia. Clin Dev Immunol 297678. 10.1155/2013/297678 PMC3572650 23424599

[26] He H, Shao Z, Liu H, Song L, Tian P, et al (2002) Immunorelated pancytopenia. Zhonghua Xue Ye Xue Za Zhi 22: 79–822. 11877054

