# Correction: Erythroblastic Islands in the Bone Marrow of Patients with Immune-Related Pancytopenia

**DOI:** 10.1371/journal.pone.0114808

**Published:** 2014-11-25

**Authors:** The PLOS ONE Staff

The first two authors, Yi-Hao Wang and Rong Fu, should be noted as contributing equally to this work.
